# Stable transformation and expression of *GhEXPA8* fiber expansin gene to improve fiber length and micronaire value in cotton

**DOI:** 10.3389/fpls.2015.00838

**Published:** 2015-10-31

**Authors:** Kamran S. Bajwa, Ahmad A. Shahid, Abdul Q. Rao, Aftab Bashir, Asia Aftab, Tayyab Husnain

**Affiliations:** ^1^Plant Biotechnology Lab, Centre of Excellence in Molecular Biology, University of the PunjabLahore, Pakistan; ^2^Plant Biotechnology, Nuclear Institute of Biotechnology and Genetic EngineeringFaisalabad, Pakistan

**Keywords:** cotton fiber, *GhEXPA8* gene, micronaire value, transformation, *Agrobacterium*

## Abstract

Cotton fiber is multigenic trait controlled by number of genes. Previous studies suggest that one of these genes may be responsible for switching cotton fiber growth on and off to influence the fiber quality produced from a cotton seed. In the present study, the *Gossypium hirsutum* GhEXPA8 fiber expansin gene was introduced into local cotton variety NIAB 846 by using an Agrobacterium-mediated gene transformation. The neomycin phosphotransferase (NPTII) gene was used as a selection marker for screening of putative transgenic cotton plants. Integration and expression of the fiber expansin gene in cotton plants was confirmed with molecular techniques including Southern blot analyses, real-time PCR. Cellulose assay was used for measurement of cellulose contents of transgenic cotton fiber. The data collected from 3 years of field performance of the transgenic cotton plants expressing GhEXPA8 showed that significant improvement has been made in fiber lengths and micronaire values as compared to control *G. hirsutum* variety NIAB 846 cotton plants. Statistical techniques were also used for analysis of fiber and agronomic characteristics. The results of this study support improvement of cotton fiber through genetic modification.

## Introduction

Cotton belongs to the Malvaceae family, which includes hibiscus and okra, and is in the *Gossypium* genus, which grows world-wide (Wendel and Cronn, 2003). Cotton crop is the main source of pure, natural cellulose fibers which is used to create fabric products. The fruit of the cotton plant is a boll that protects the seeds and cotton fibers, which are soft and delicate (Constable et al., 2014). Cotton (*Gossypium* spp.) is one of the most important economic crops in the world, especially *Gossypium hirsutum* L., which provides more than 90% of the total cotton fiber produced. The fibers are the seed hairs of cotton, originating from the epidermal cells of the ovular surface (Liu et al., 2005). Cotton bolls are open at maturity, revealing soft masses of fibers. Cotton fibers are long (30–40 mm) and thin (15 μm) unicellular structures that emerge from the epidermal cells in the outer integuments of cotton ovules (Kim and Triplett, 2001).

Cotton fiber development completes in four distinct stages: fiber initiation, elongation, secondary cell wall biosynthesis, and maturation. These stages overlap with each other during the formation of a mature fiber. Fiber development is varied among the different types of cotton species, for example, lint fiber and fuzz fiber form at different times, before and after anthesis, in *G. hirsutum* (Haigler et al., 2005). The time required for the development of a cotton boll from a fertilized flower is approximately 45–50 days. The development of a cotton boll has three stages: boll swelling, boll filling, and boll bursting (Ruan et al., 2001).

The mechanisms involved in ovule epidermal cell development result in the production of cotton fiber, which is used to manufacture textiles (Pu et al., 2008). Thus, there is a demand for improved cotton fiber in textiles, and the developmental changes in the fiber may affect its quality parameters. The manipulation of the cotton fiber quality parameters potentially impacts on fiber length (the average length of the longer one-half of the fibers), micronaire value [fineness (linear density) and maturity (degree of cell-wall development)], and fiber strength (force necessary to break the beard of fibers; Seagull et al., 2000).

The molecular genetics of multigenic families provide new insights into transcription and expression signatures to better understand cotton fiber growth and development (Boopathi and Ravikesavan, 2009). The role of genes in any specific stage of development depends on their function and other related genes with similar functions. The major and minor isoforms of gene family members are expressed at different levels depending on the specific developmental stage of the fiber cell. The expression of expansin genes up regulates fiber cell expansion (Vogler et al., 2003; Zhang et al., 2008).

A number of genes are required, which are differentially expressed during different stages of fiber development (Wang et al., 2010). Only a few of the genes involved in the biosynthesis of many fiber-specific structural proteins, enzymes, polysaccharides, waxes, or lignins have been identified (Li et al., 2002). These fiber-specific genes may be potential targets for cotton fiber improvement (Hovav et al., 2008; Rapp et al., 2010).

Advanced technological developments are innovating many of the aspects of basic and applied plant transgenic science. Plant genetic engineering has provided new methods to modify crops and new solutions to solve specific needs. The development of cell biology procedures to regenerate plants from single cells or organized tissue, and the use of genetic engineering for crop modification is correlated with the discovery of novel techniques to transfer genes to plant cells. Plant transformation technology has become an adaptable platform for crop improvement and for studying gene function. New plant transformation vectors, methodologies, and techniques have improved the efficiency of transformation, making the stable expression of transgenes in plants achievable (Rao et al., 2009).

The characterization of the genes involved in fiber development is a prerequisite for their modification. *EXPANSIN* is one the important gene families involved in fiber elongation and expansion. The *EXPANSIN*s have been investigated for long in the expansion of cells. The fiber specific *EXPANSIN*s have been reported and shown to contribute partly in fiber expansion (Indrais et al., 2011). It has been reported that the regulation of cell wall extensibility during cell expansion is controlled, in part, by expression of EXPANSIN genes in cotton (Arpat et al., 2004). EXPANSINs have been reported not only to increase cell size but also the fruit size (Cosgrove, 2000). Expression of EXPANSIN genes has also been observed during fiber elongation (Ruan et al., 2001). Given the inferred importance of EXPANSIN genes in cotton fiber elongation and the association of multigene EXPANSIN family members in the expanding cells of other plant species (Cosgrove, 2000; Li et al., 2005), it was reasonable to hypothesize that some EXPANSIN genes could be involved in the development of cotton fibers. Expansins are a family of closely related non-enzymatic proteins in the plant cell wall that have important functions associated with cell growth, fruit softening, abscission, emergence of root hairs, pollen tube invasion of the stigma and style, meristem function, and cell wall loosening (Cosgrove, 2000). In this study, hypothesis was over expression of EXPANSIN gene in cotton genome for fiber characteristics improvement through the *Agrobacterium*-mediated transformation of *GhEXPA8*; into local cotton variety NIAB 846 was performed. Different molecular techniques were used for confirmation of transgenic cotton plants. These plants demonstrate significant improvement of fiber quality as compared with the control plants.

## Materials and methods

### Selection and stable transformations of cotton varieties

Fifteen local cotton varieties, specifically, CIM 497, NIAB 846, CIM446, CIM499, CIM473, CIM 443, BH 118, BH 75, BH 79, BH 95, BH 557, MNH 93, NIAB 78, FH 672, and FH 673, were screened for transformation on the base of germination (%) in soaking experiment, as previously shown by Rao et al. (2006). Local cotton variety such as NIAB 846 was selected for transformation because higher rate of germination (%) in soaking experiment. One day before the transformation experiment, a bacterial culture with the *GhEXPA8* gene of interest was started in YEP broth (Yeast Extract Peptone Medium) on a 30°C shaker. Cotton seeds of the *G. hirsutum* var. NIAB 846 were soaked in an autoclaved flask of water, in the dark, at 30–37°C for 48 h. On the day of experiment, the bacterial culture with the *GhEXPA8* gene of interest was harvested and dissolved in MS medium. Mature embryos were isolated from germinating seeds and the apex of the shoot was cut with a sterilized blade. Then, the embryos were co-cultivated for 1–2 h with the Agrobacterium strain LBA-4404 containing the *GhEXPA8* gene. The embryos were dried on sterilized filter paper and transferred to plates with MS medium (Murashige and Skoog, 1962) for 2–3 days at 28°C in a condition-controlled room. After 2–3 days, the embryos were subcultures in tubes containing MS medium with kanamycin as a selection medium. Every 15 days, the transgenic cotton plants were subcultures into new test tubes. After 30–45 days of selection on the kanamycin medium, putative transgenic plants were transferred onto shoot and root regeneration media without kanamycin. After 2 months, the healthy, putative transgenic cotton plants were shifted to pots containing loamy soil. Acclimatization of transformed plants was performed. The stable, putative transgenic plants were subjected to molecular analysis after 15–20 days of shifting (Hussain et al., 2005; Bajwa et al., 2013).

### Detection and integration of the *GhEXPA8* gene in putative transgenic cotton plants

Newly formed leaves from transgenic cotton plants with the fiber transgene and from control plants were used to extract genomic DNA according to Saha et al. (1997), with some modifications. PCR was used to confirm the presence of the *GhEXPA8* fiber transgene in putative transgenic cotton plants as a 960 bp PCR product with primers designed from the promoter region and the gene region (Cronn et al., 2002). The pairs of primers were used for the analysis of transgenic cotton plants at T_0_, T_1_, and T_2_ generations were present as a Supplementary Data (Supplementary Table 1). The reaction conditions were as follows: an initial denaturation at 95°C for 3 min; 35 cycles of denaturation at 94°C for 45 s, annealing at 49°C for 45 s (T_0_ generation), 53°C for 30 s (T_1_ generation), 51°C for 30 s (T_2_ generation), and extension at 72°C for 45 s; and a final elongation step at 72°C for 10 min. The stable integration and presence of *GhEXPA8* in the plant genome was confirmed using Southern blot analysis. Southern blot analysis was performed from genomic DNA extracted from confirmed transgenic cotton plants as described by Anklam et al. (2002)and Rao et al. (2006). Stable integration was detected with a *GhEXPA8* gene-specific probe after the genomic DNA was digested with an EcoR1 restriction enzyme.

### Confirmation of transgenic plants with NPTII, 35SCaMV, and VirG primers

For confirmation of the transgenic nature of the cotton plants, PCR was performed for the amplification of NPTII (marker gene), 35SCaMV (promoter gene), and VirG (virulence gene) on the base of genomic DNA extracted from transgenic cotton plants (Anklam et al., 2002; Sundar and Sakthivel, 2008). The sequences of the primer pairs were present in Supplementary Data (Supplementary Table 1). The reaction mixture of PCR was as follow; 20 μl reaction mixture volume containing 1X reaction buffer, 15 ng DNA templates, 1.5 mM MgCl_2_, 1 mM of each dNTPs, 1 pmole of each primer, and 1 unit of Taq DNA polymerase (Fermentas). PCR was carried out in a thermal cycler using the following conditions: initial denaturation at 95°C for 3 min; 35 cycles of denaturation at 94°C for 45 s, annealing at 54°C (for NPTII), 52°C (for 35SCaMV) or 53.9°C (for VirG) for 45 s, extension at 72°C for 45 s; and a final extension at 72°C for 10 min. The amplified DNA fragments were electrophoresed on a 1.5% (w/v) agarose gel (Anklam et al., 2002; Sundar and Sakthivel, 2008).

### Quantitative real time expression of transgenic cotton plants

Newly emerged young leaves of transgenic and a control plant was used for the extraction of RNA according to the procedure performed as described by Jaakola et al. (2001). Currently, the standard protocol to measure mRNA expression that offers the best sensitivity, dynamic range, and reproducibility is quantitative real-time PCR. Using qRT-PCR (Maxima SYBR Green/ROX qPCR Master Mix (2X), Catlog # K0221, Thermo Scientific, USA). oligo (dt) primers were used for conversion of complementary DNA from mRNA transcripts. The complementary DNA of *GhEXPA8* fiber expansin gene was then exponentially amplified with PCR using gene-specific primers. The concentration of the amplicon was monitored with SYBR Green dye. The endogenous expression of glyceraldehyde 3-phosphate dehydrogenase (GAPDH) was used as the internal control. All PCR reactions were performed with the following conditions: 94°C for 3 min and 40 cycles of 45 s at 94°C, 45 s at 52°C, and 45 s at 72°C. Each qRT-PCR sample had three replicates (Rao et al., 2011).

### Extraction of cellulose from cotton fiber

The fiber was digested with an acetic-nitric reagent and an anthrone reagent, and the amount of cellulose was measured with a spectrophotometer as described by Updegraff (1969). This method is used for the removal of impurities, such as lignin, hemicellulose, and xylosan, from cotton fiber with the help of acetic acid and nitric acid. Anthrone dissolved in sulphuric acid was used to extract the cellulose from the cotton fiber. The resulting colored compound was assayed with a spectrophotometer at a wavelength of approximately 630–635 nm according to the procedure (Shu et al., 2009).

### Estimation of quality parameters

Cotton samples during the boll opening period were harvested from the six transgenic lines and the control plants at the T0 (2009), T1 (2010), and T2 (2011) generations. The fiber quality tests were performed at the Test Centre of Cotton Fiber Quality at the APTMA in Pakistan (ICC standard). Data were collected on fiber qualities such as fiber length, fiber strength, fiber uniformity, and micronaire value [fineness (linear density), and maturity (degree of cell-wall development)]. Every data point represents three replicate samples (Bajwa et al., 2013). The increase in cotton fiber length was determined by the mean of the fibrogram using a fibrograph. The fibrogram span lengths measure the length of fiber at 50% and 25%. The cotton fiber strength (lbs PSI) and micronaire value of the cotton fiber were calculated with high volume instruments (HVI). The uniformity index was determined with mean length (ML) and upper-half mean length (UHML) using the following formula:

UNIFORMITY  INDEX=ML÷UHML×​100

### Measurement of quantitative characters

The measurement of quantitative parameters is important for the performance of a crop in field conditions. A variety of agronomic characteristics were measured, including the number of bolls, boll weight per plant, ginning out turn (GOT %), and average yield. The most important morphological data, including the yield of putative transgenic and control plants, were calculated during T_0_, T_1_, and T_2_ generations.

### Statistical analysis

Two well-reputed methods, Fisher's analysis and Co-Stat, were used to determine the analysis variance of the data. The least significant difference test (LSD) at 5% probability was used to compare the experimental and control plants (Petersen, 1994). Fiber length, fiber strength, and fiber micronaire values of the transgenic cotton were calculated using the classical fiber quantitative analyses of boll weight per plant, seed weight per boll, ginning out turn percentage and average yield. Statistical analysis was also used for phenotypic and genotypic correlation of transgenic cotton plants. Detailed procedure of phenotypic and genotypic correlation was present as Supplementary data.

## Results

### *Agrobacterium*-mediated transformation of cotton variety NIAB 846

**Figure 1:**
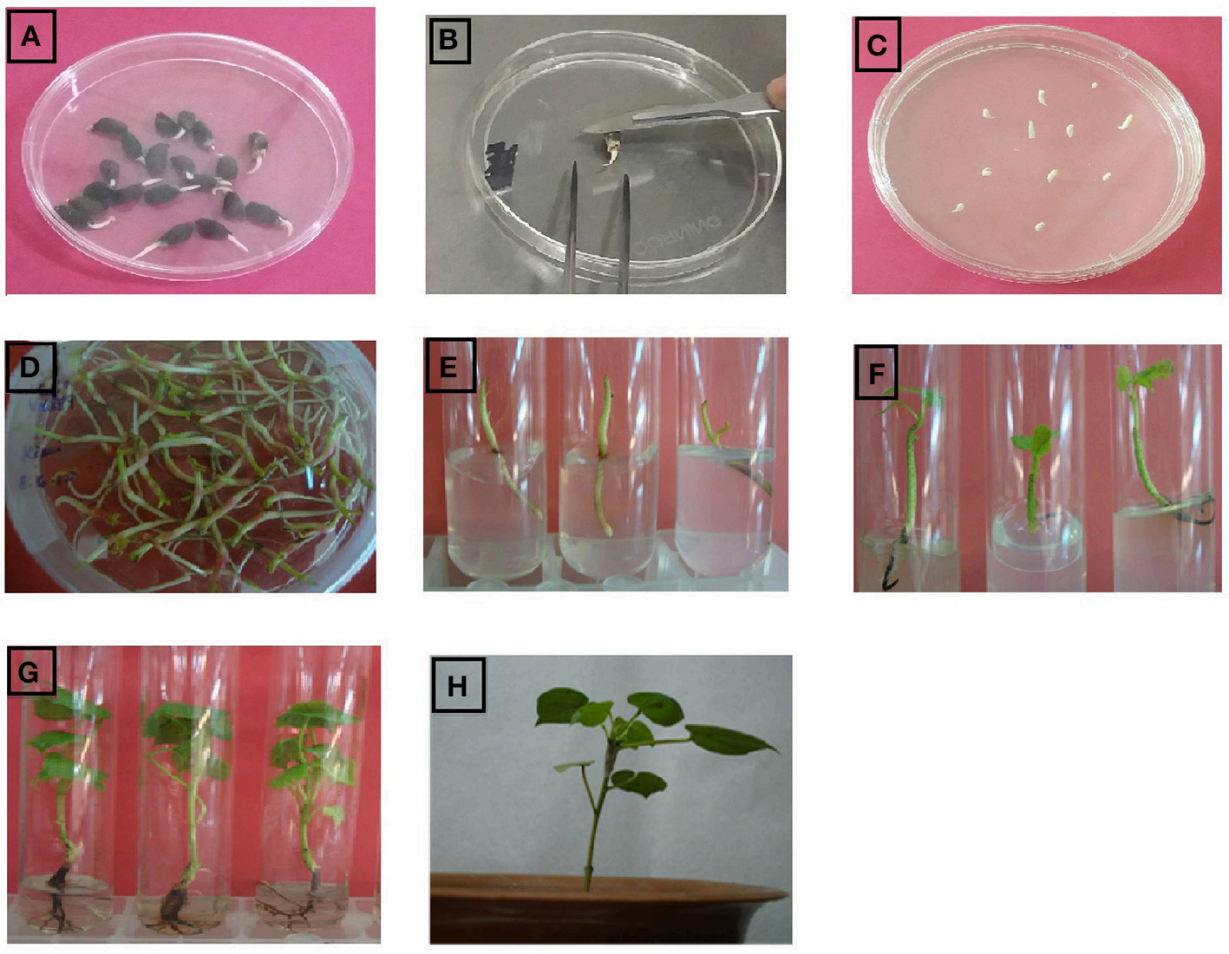
**Complete protocol of agrobacterium mediated transformation in cotton plant embryos. (A)** germinated cotton seeds, **(B)** isolation and injuring of cotton embryos, **(C)** Agrobacterium treated embryos on MS medium, **(D)** Agrobacterium treated embryos on MS medium after 3 days, **(E)** implantation of cotton embryos in test tube containing MS medium with kanamycin, **(F)** shoot growing on MS medium containing kanamycin selection, **(G)** root development on MS rooting medium, **(H)** transgenic cotton plants shifted to soil for acclimatization.

*Agrobacterium*-mediated transformation was used to incorporate the *GhEXPA8* gene into NIAB 846. A total of 8500 embryos were transformed with the *GhEXPA8* gene and selected on MS medium containing 50 mg/ml kanamycin (Figure 1). There were 106 putative transgenic plants, an efficiency of 1.24% of 8500 transformed embryos on the base of transformation procedure and on the base of confirmed transgenic cotton plants is 0.07%, obtained and transferred to MS medium with kanamycin selection. The putative transgenic plants were screened through selection medium as shown in (Figures 1D,E). On the base of PCR analysis of transgenic cotton plants at T_0_ and T_1_ generations, these transgenic cotton plants were subjected to molecular analysis such as PCR, southern blot analysis, Real Time PCR, cellulose assay, fiber characteristics and yield parameters at T_2_ generation in order to protect chimeric presence of gene in few part of cotton plants. There was only one cause behind this protocol in order to screen the transgenic cotton plants from chimera or low expression of gene.

### Identifying the *GhEXPA8* fiber gene in cotton plants

**Figure 2:**
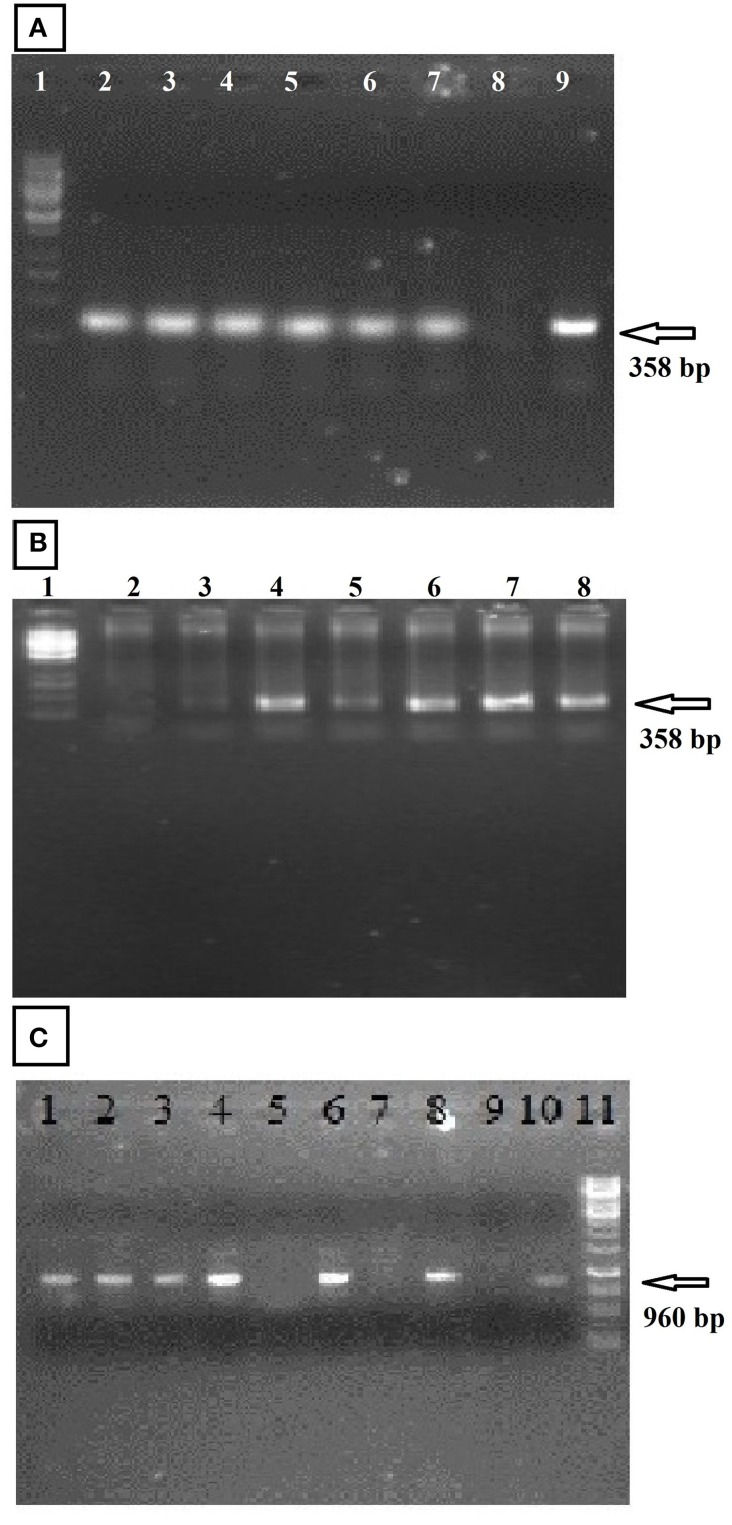
**PCR amplification of transgenic cotton plants during three generations (Fiber Gene *GhEXPA8*)**. **(A)** represented the PCR amplification of transgenic cotton plants at T_0_ generation, lane 1: 1 kb DNA marker, lane 2–7: transgenic cotton plants, Lane 8: Negative Control, Lane 9: Positive Control; **(B)** PCR amplification of transgenic cotton plants during T_1_ generation, lane 1: 1 kb DNA marker, lane 2: negative control, lane 3–8: transgenic cotton plants; **(C)** During T_2_ generation Lane 1–8 show putative transgenic plants in which lane 1, 2, 3, 4, 6, and 8 show positive transgenic plants having gene amplification from orientation primers resulting in band of 960 bp whereas, Lane 5 and 7: untransformed cotton (negative amplification), Lane 9: DNA from untransformed cotton (Negative Control), Lane 10: DNA from pGA482-*GhEXPA8* (Positive Control), lane 11:1 kb DNA ladder Whereas, the arrow indicate the detection of gene in transgenic cotton plants.

PCR of the putative transgenic plants confirmed that at least six plants contained the *GhEXPA8* gene. The gene and promoter region primers 5′-TGTGAGTAGTTTCCCGATAA-3′ and 5′-ATCCTTCCTTGTCTTCCTC-3′ were successfully amplified as a 960 bp fragment in T_2_ generation (Figure 2C) and primer sequence 5′-CCCCCTAACTATGCTTTATC-3′ and 5′-ATTTGTAGAGAGAGACTGGTGA-3′ was used for the analysis of transgenic cotton plants during T_0_ (Figure 2A) and T_1_ (Figure 2B) generations. A total of six plants of the 106 putative transgenic cotton plants contained the transgene. No amplification was detected in the negative control plants (Shehzad, 2013).

### Confirmation of transgenic plants with NPTII, 35SCaMV, and VirG primers

**Figure 3:**
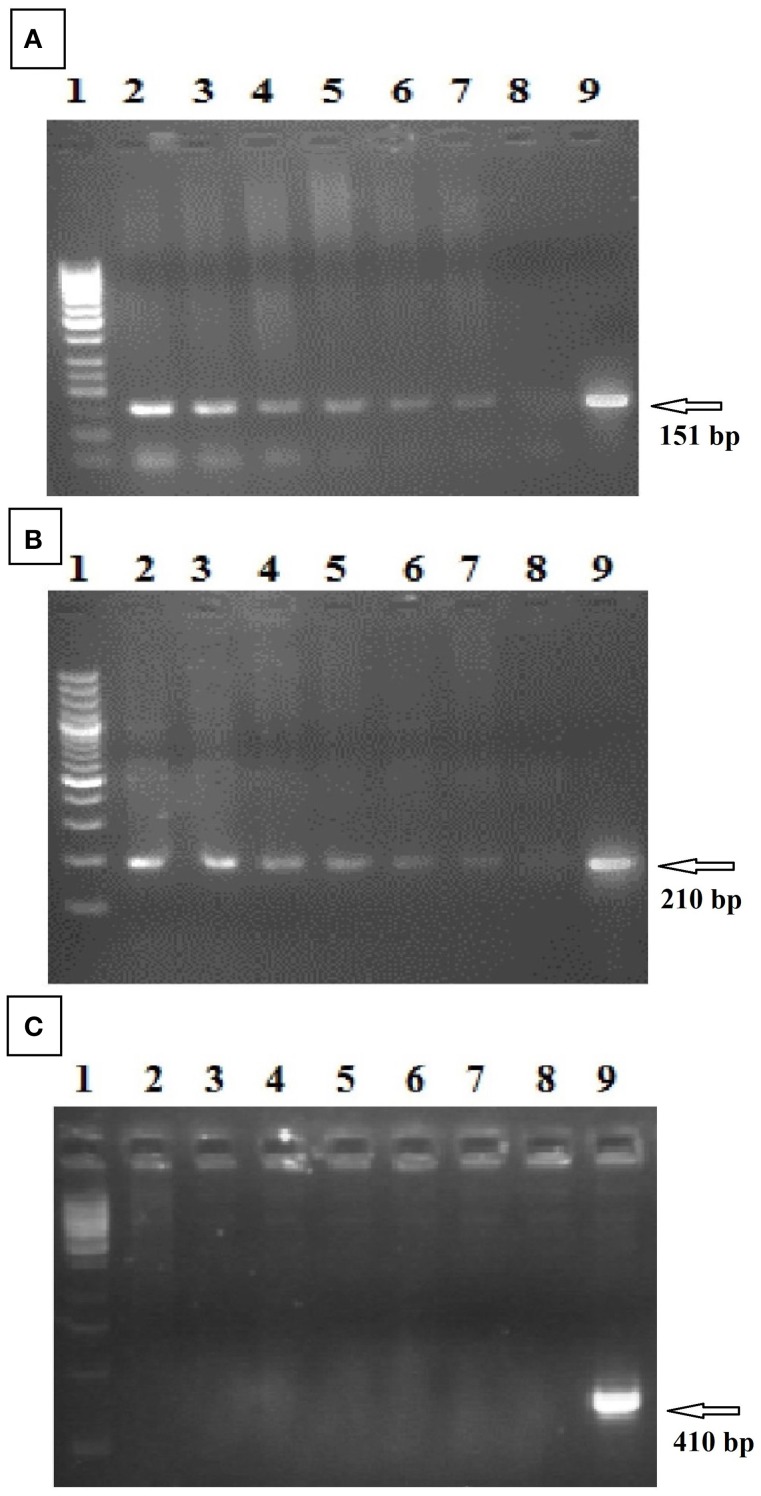
**PCR analysis of transformed cotton for NPTII gene, 35S CaMV promoter region, and VirG gene**. **(A)** (NPTII gene): Lanes 2–7: putative transgenic cotton lines; lane 8: untransformed cotton, lane 9: pGA482-*GhEXPA8*; lane 1: 50 bp DNA ladder (Fermentas, USA). **(B)** (35S CaMV promoter region): lane 1: 100 bp DNA ladder (Fermentas, USA) Lanes 2–7: putative transgenic cotton lines; lane 8: untransformed cotton, lane 9: pGA482-*GhEXPA8*. **(C)** (VirG gene of *Agrobacterium*): lane 1: 1 kb DNA ladder (Fermentas, USA) Lanes 2–7: putative transgenic cotton lines; lane 8: untransformed cotton, lane 9: pGA482-*GhEXPA8*.

To identify the fiber expansin gene (GhEXPA8) in NIAB 846, amplification of a 151 bp PCR product for the NPTII gene (kanamycin resistance gene) and a 210 bp PCR product for the 35S promoter region was performed using specific primers. In addition, PCR was performed for the VirG gene (Agrobacterium) with specific primers. Figures 3A,B show the 151 bp amplification product of the NPTII gene and the 210 bp product of 35S. The VirG primers did not result in amplification with PCR (Figure 3C). Untransformed cotton plants were negative for all NPTII, 35S CaMV and VirG genes (Bajwa et al., 2014).

### Integration of the *GhEXPA8* gene in cotton plants

**Figure 4:**
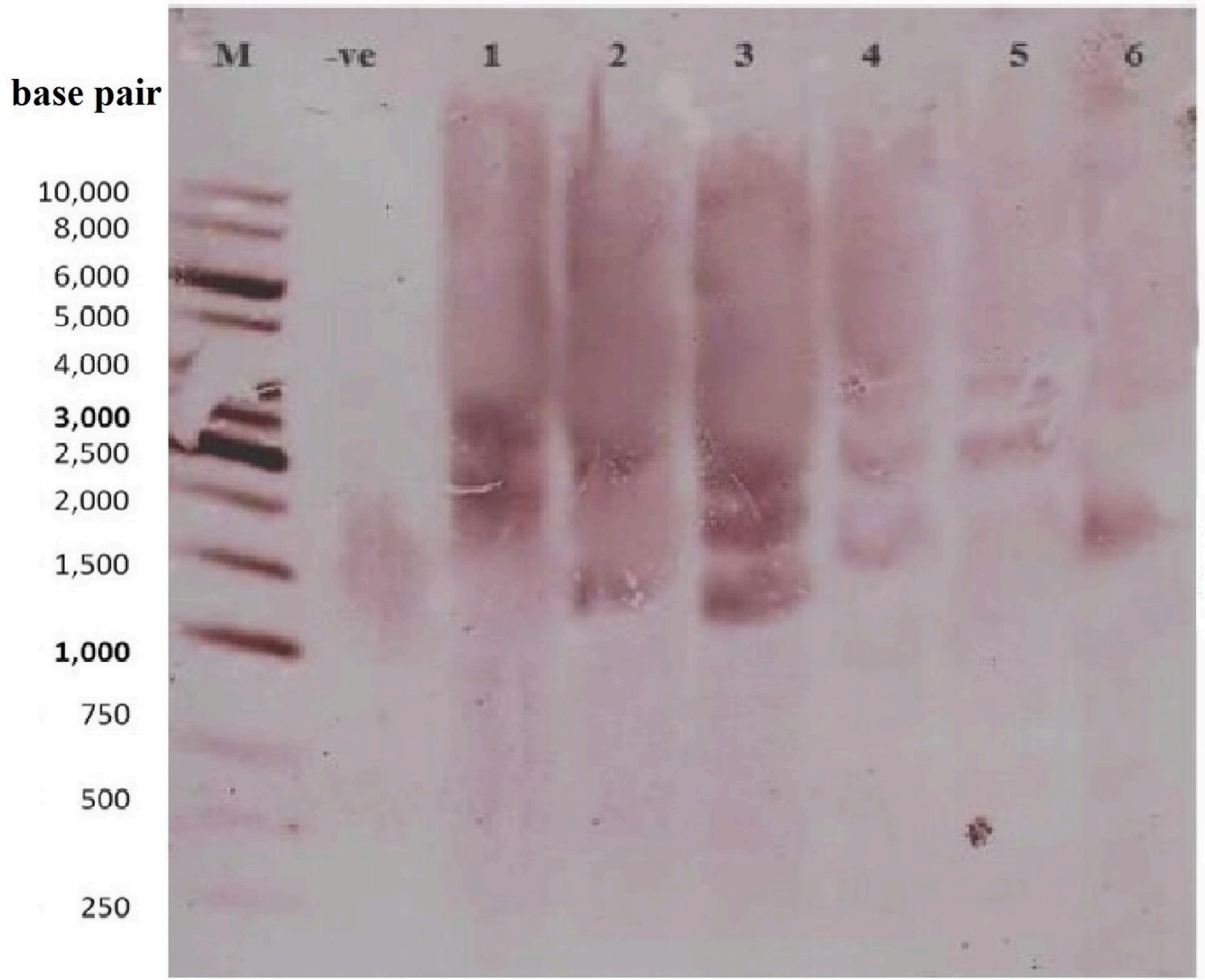
**Determination of copy number of transgene (Fiber Gene *GhEXPA8*) through southern blot analysis**. Lane M: 1 kb DNA Ladder, Lane 1: Negative Control (DNA of untransformed plant), Lane 2–7 (FG-8-1, FG-8-4, FG-8-5, FG-8-11, FG-8-13, and FG-8-15 respectively): Selected Transgenic Cotton Plant Samples (pGA482-*GhEXPA8*) were digested with EcoR1, probed with biotin-labeled *GhEXPA8* DNA to assess copy number of the transgene in the transformed plants. The number of bands revealed that the plant FG-8-15 had one copy of the transgene in its genome, FG-8-1, FG-8-4, and FG-8-13 had two copies of the transgene in its genome, whereas the plants FG-8-5 and FG-8-11 had three copies of the transgene. Figure determined 1.56 kb highlighted region along with other highlighted region which gives information about the copy number. Lane 1 contains 1 Kb ladder, lane 2 represented negative controls, in lane 3–8 indicate the integration of fiber gene *GhEXPA8* in selected transgenic cotton plant samples.

Southern blot analysis was used to determine copy number of *GhEXPA8* expansin gene into the cotton plant genome. A *GhEXPA8*-specific probe identified the fiber gene copy numbers in the cotton genome after the genomic DNA of putative transgenic plants were digested with EcoR1 restriction enzyme (Figure 4). In this figure, lanes 3, 4, 7, and 8 show two copies and lanes 5 and 6 show three copies of the *GhEXPA8* fiber gene integrated into the genomic DNA of putative transgenic cotton plants.

### Expression level of fiber gene in T2 transgenic plants

**Figure 5:**
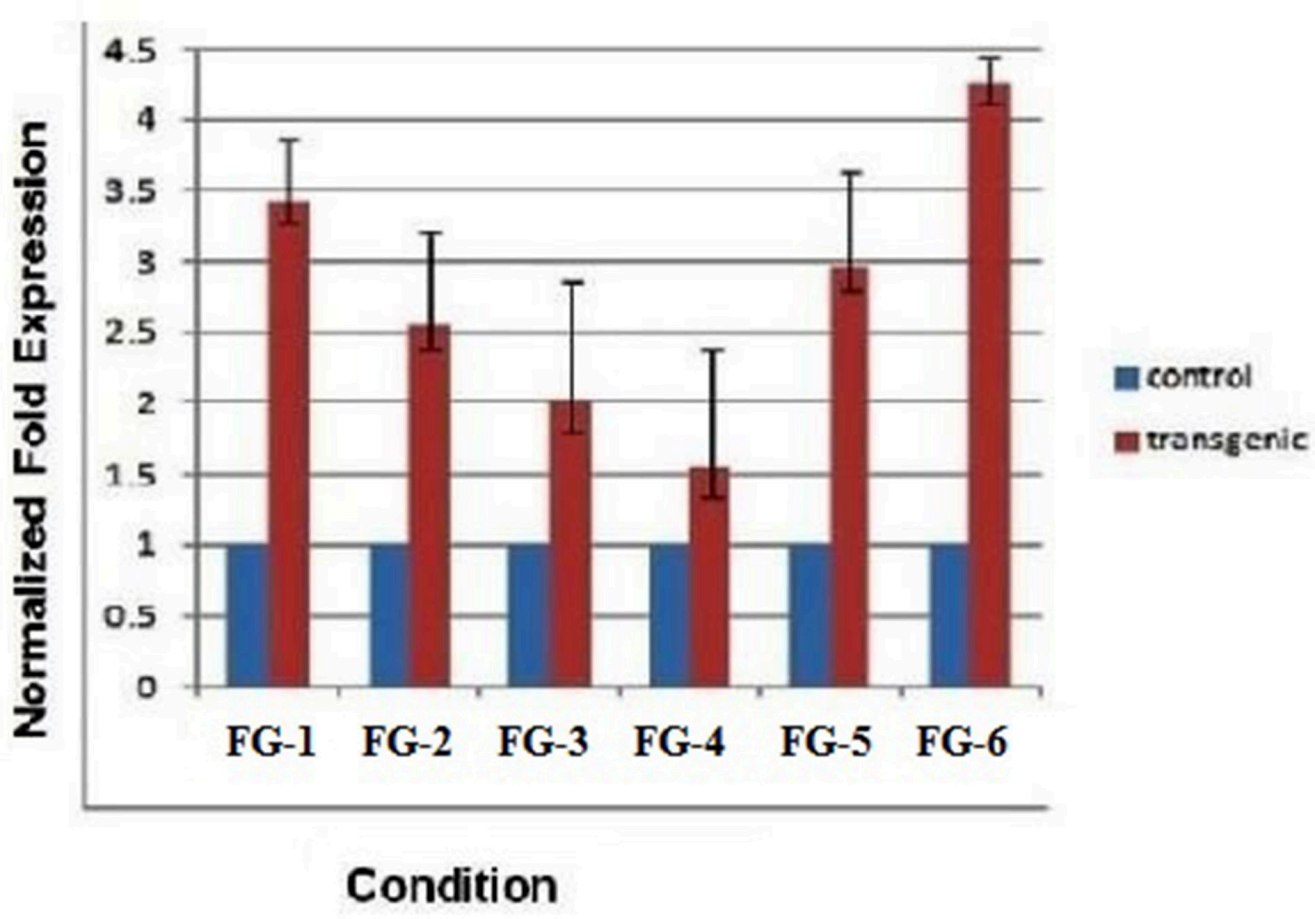
**Quantitative real time PCR to determine the expression of transgenic cotton plants (Fiber Gene *GhEXPA8*)**. Lines FG-8-1, FG-8-4, FG-8-5, FG-8-11, FG-8-13, and FG-8-15: described the different expression levels of transgenic and control mRNA samples, blue color represent the expression level of control plant samples (mRNA of untransformed cotton), red color indicated the mRNA expression level of putative transgenic lanes, GAPDH was used as an internal control for normalization.

The quantitative measurement of mRNA expression in leaf samples from 6 plants transformed with the *GhEXPA8* fiber gene was performed with qRT-PCR. The *GAPDH* gene was used as the reference gene for normalization. Figure 5 shows the variation in gene expression of the six transgenic lines of T2 generation. Plant line *GhEXPA8-1* and plant line *GhEXPA8-15* produced the 2.5- and 3.5-fold expression of expansin gene in T2 of transgenic *G. hirsutum* as compared to control which was highest among all transgenic lines. Plant line *GhEXPA8-4* and plant line *GhEXPA8-13* were less expressive (1.5-fold and 2-fold, respectively) when compared to the control line. Plant line *GhEXPA8-5* and plant line *GhEXPA8-11* showed the least expression of transgene (1-fold and 0.5-fold, respectively). The expression level of plant line *GhEXPA8-11* was much lower compared to the other transgenic lines. Plant lines *GhEXPA8-1* and *GhEXPA8-15* had excellent fiber quality parameters as shown in Figure 5 whereas, bars represent the variation among replicates.

### Cellulose assay

**Figure 6:**
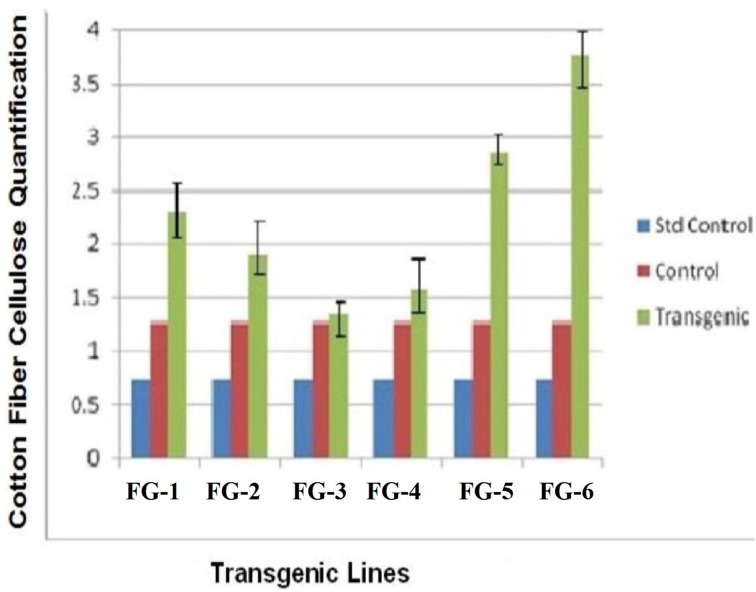
**Comparison of cellulose contents of transgenic and control cotton fiber along with standard control**. Plants in groups 1 to 6 were showed three different samples of cellulose. Blue color represent the amount of cellulose from Avicel PH-101pure cellulose, red color represent the amount of cellulose obtained from untransformed cotton, green color indicated the depicts the presence of cellulose in transgenic cotton.

The quantity of cellulose in the cotton fibers after genetic transformation were increased from 20 days post-anthesis (DPA), with improved changeability between the transgenic and parental cotton fibers. In this study, we compared the cellulose content of transgenic cotton fibers and control cotton fibers. Plant line 6 (GhEXPA8-15) produced 3-folds more cellulose, plant line 5 (GhEXPA8-13) produced 2.5-fold more cellulose, plant line 1 (GhEXPA8-1) produced 2-fold more cellulose, and plant line 2 (GhEXPA8-4) produced 1.5-fold more cellulose than the control lines. The cellulose levels of two transgenic lines, 3 (GhEXPA8-5) and 4 (GhEXPA8-11), were slightly higher than the control line, but lower than the other transgenic lines. The transgenic plant lines 1, 4, 5, and 6 had higher cellulose content than the control line, which positively affected one or more fiber quality parameters (Figure 6).

### Analysis of fiber quality parameters

After the critical examination of 106 transgenic cotton plants with the *GhEXPA8* fiber gene, we determined consistent improvement in cotton quality characteristics. Transgenic cotton plants were evaluated for quality, and six transgenic cotton plants were selected for their expression of the transgene over three generations (2009–2011). A *G. hirsutum* NIAB 846 parent was used as a control for comparison. Fiber samples were collected from each transgenic plant and sent to Cotton Research Station Multan (CRS) and APTMA Pakistan for fiber testing using HVI.

**Figure 7:**
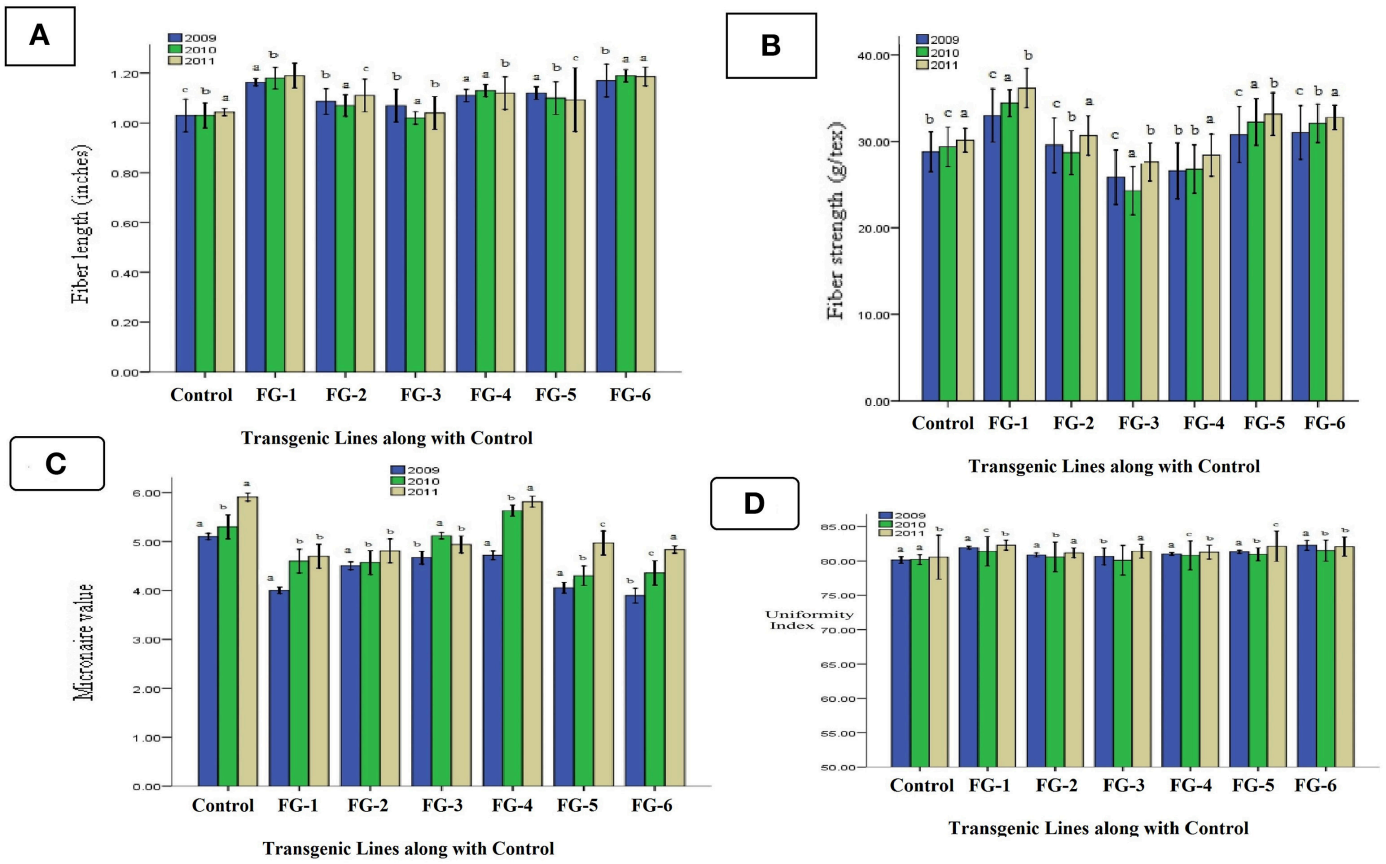
**Comparison of fiber length, fiber strength, fiber micronaire value, and fiber uniformity index of transgenic cotton plants with control (T0, T1, and T2):** fiber length (**A**), fiber strength (**B**), fiber micronaire value (**C**), and fiber uniformity index (**D**) of different transgenic cotton plants T_0_, T_1_, and T_2_ in generations of 2009, 2010, and 2011, blue color represent fiber analysis at T0 generation, green color indicated fiber analysis at T1 generation, brown color highlight fiber analysis at T2 generation. Note: Bar Determined the Standard Error.

The evaluation of fiber qualities in transgenic cotton plants indicated positive effects of the transgene in all three generations (T_0_, T_1_, and T_2_). Three important fiber quality parameters were improved: fiber length, micronaire value (mike value) and fiber strength. Fiber length increased 17% (1.18 inches) in the transgenic line *GhEXPA8-15*. The fiber length of transgenic line *GhEXPA8-1* increased 20% (1.20 inches). Transgenic lines *GhEXPA8-4* and *GhEXPA8-13* improved 10% (1.12 inches), and the remaining two transgenic lines had a lower response rate (Figure 7A). The second important fiber quality parameter is fiber strength, which increased in the six transgenic lines in all three generations. After the genetic transformation of the *GhEXPA8* fiber gene, fiber strength was improved 17% (34.53 g/tex) in line *GhEXPA8-1*, 10% (33.18 g/tex) in line *GhEXPA8-15*, and 9% (32.06 g/tex) in line *GhEXPA8-11* (Figure 7B). The micronaire values of the transgenic cotton plants were finer than the parental plants, and improved 17.81% (4.43) in line *GhEXPA8-1*, 19.10% (4.36) in line *GhEXPA8-15*, 17.62% (4.44) in line *GhEXPA8-13*, and 14.10% (4.63) in line *GhEXPA8-4* (Figure 7C). The uniformity index (%) of transgenic plant lines *GhEXPA8-1* and *GhEXPA8-15* were superior to control plants (Figure 7D). In this Figures 7A–C represents triplicate replication within treatment and also indicated the significant improvement of gene in transgenic cotton plants. a; most significant, b; significant and c; least significant. The level of confidence of statistical analysis in this study is 5%.

### Statistical analysis of fiber quality characteristics

After the significant analysis of the putative *GhEXPA8*-transgenic cotton plants, the data show a correlation between fiber length and micronaire value. For example, if the fiber length of a transgenic plant was improved, then the micronaire values were also significantly affected. From the results of fiber length indicated LSD values 0.048 (2009), 0.017 (2010) were greater than all difference of mean value from control but less than control in 0.10 (2011). The same subscripts on bar determines that the values differ significantly in 2009 and 2010 but do not differ significantly in 2011 at *P* > 0.05 level (significant error). Statistical data of fiber strength represented LSD values 0.057 (2009) was less than all difference of mean values from control but 0.011 (2010) and 0.023 (2011) was greater than all difference of mean values from control. The difference of subscripts on bar determines that the values differ not significantly in T0 generation (2009) but differ significantly at *P* > 0.05 level in T_1_ and T_2_ generations (2010, 2011). Statistical analysis of fiber micronaire value showed LSD value 0.021 was greater than all difference of mean values from control. The difference of subscripts on bar determines that the values differ significantly at *P* > 0.05 level in all three generations like T_0_, T_1_, and T_2_ (2009, 2010, 2011) and statistical analysis of fiber uniformity index indicated that LSD 0.032 (2009, 2011) were greater than all difference of mean values from control but 0.18 (2010) was less than all difference of mean values from control. The difference of subscripts on bar determines that the values differ significantly at *P* > 0.05 level in T_0_ and T_2_ generations (2009, 2011) and not significant in T_1_ generation (2010).

### Mendelian segregation analysis

After T_2_ generation of transgenic cotton plants, law of Mendelian segregation was performed for the analysis of fiber characteristics (Fiber length, fiber strength, fiber micronaire values, and fiber uniformity index). Calculations and conclusions by looking at the number of cotton plants that showed improvement in fiber characteristics, the T_2_ generation will bring plants that have improved fiber characteristics because it is integrated *GhEXPA8* fiber expansin gene on the cotton genome. For plants that have low expression *GhEXPA8* fiber expansin gene in their genome such as FG-8-5, FG-8-11, these plants showed little improvement in fiber characteristics (Table 1).

**Table 1:** **Analysis of variance (ANOVA) of fiber characteristics such as fiber length, fiber strength, fiber micronaire value and fiber uniformity index of transgenic cotton plants with ***GhEXPA8*** fiber expansin gene**.

		**Sum of Squares**	***df***	**Mean Square**	***F***	**Sig**.
Micronaire Value	Between Groups	4.235	6	0.706	2.059E3	0
	Within Groups	0.005	14	0		
	Total	4.240	20			
Strength	Between Groups	156.872	6	26.145	1.389E3	0
	Within Groups	0.264	14	0.019		
	Total	157.135	20			
Length	Between Groups	0.053	6	0.009	11.0039	0
	Within Groups	0.001	14	0		
	Total	0.055	20			
Uniformity	Between Groups	5.393	6	0.899	1.066E3	0
	Within Groups	0.012	14	0.001		
	Total	5.405	20			

Chi Square (X^2^) test was used for analysis of 3 years data of fiber characteristics, while the results of this analysis as follows: FG-8-1 has *F*-value 0.2 and *F* Table 3.84, so H0 is rejected and obtained Mendelian ratio is 3:1. In line FG-8-4 has an *F*-value 0.555 and *F* Table 3.84, H0 is rejected so that the conclusions obtained Mendelian ratio of 3:1. FG-8-13 has an *F*-value of 0.0222 and the *F* Table 3.84 so H0 is rejected, resulting in a 3:1 Mendelian ratio. The last Chi Square test on line FG-8-15 has an *F*-value 1.0888 and *F* Table 3.84 so H0 is rejected, so it can be concluded Mendelian ratio is 3:1.

### Quantitative characteristics of transgenic cotton plants

**Figure 8:**
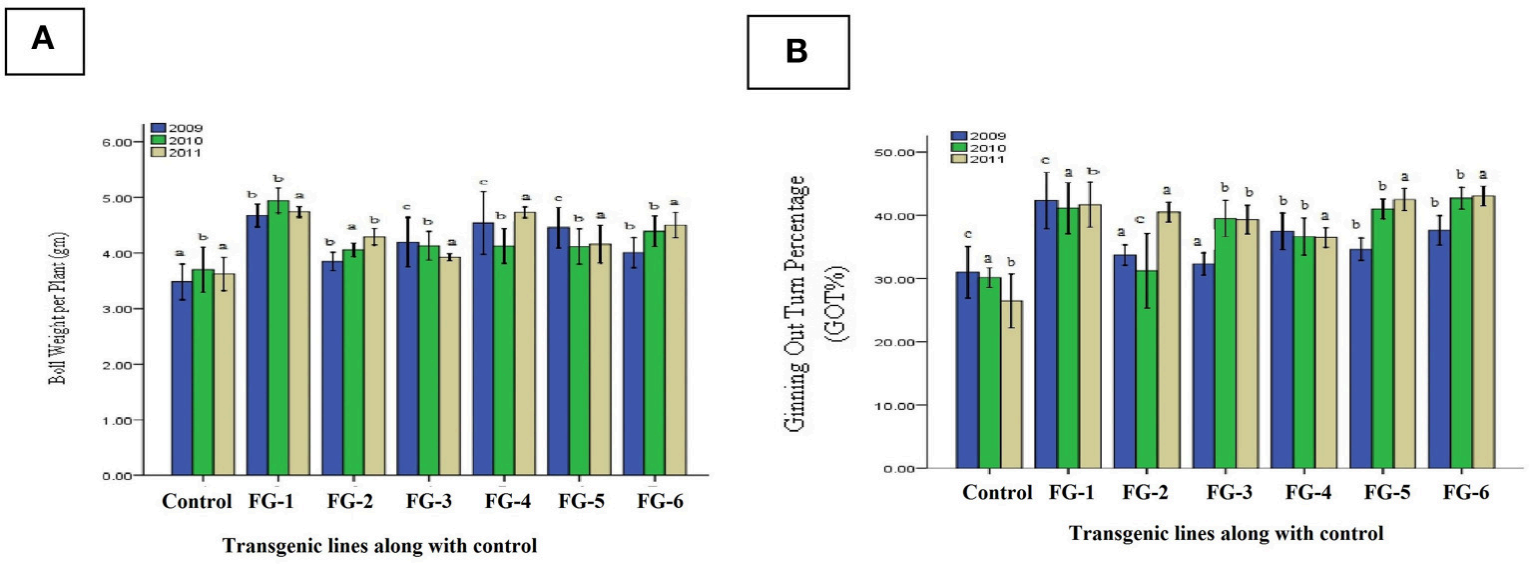
**Comparison of boll weight per plant and ginning out turn percentage (GOT%) of transgenic plants**. **(A)** indicate the Boll Weight per Plant and **(B)** represent the Ginning Out Turn percentage (GOT%) of putative transgenic cotton plants with *GhEXPA8* fiber gene for T_0_, T_1_, and T_2_ generations in year 2009, 2010, and 2011, blue color represent morphological analysis at T0 generation, green color indicated morphological analysis at T1 generation, brown color highlight morphological analysis at T2 generation, Note: Bar determined the standard error.

In the field, crop evaluations are also based on quantitative parameters such as boll weight per plant, ginning out turn percentage and yield, in addition to the quality characteristics. We analyzed the transgenic cotton plants for their delinted seed weight, fiber weight per seed and fiber ginning out turn percentage. We observed the following improvements in these characteristics: 21% (0.113 g) increase in the delinted seed weight (Figure 8A); 27% (0.061 g) increase in the fiber weight per seed (Figure 8A); and 36% increase in the fiber ginning out turn percentage (Figure 8B). Likewise, other agronomic parameters such as the number of bolls, plant height and average yield were greatly improved. In general, there were substantial differences between the transgenic and control cotton plants, which may be a highly attractive product for textile communities in Pakistan because farmers can cultivate cotton not only for quantity, but quality. From the statistical analysis of boll weight per plant indicated Least Significant Difference value 0.004 was greater than all difference of mean values from control. The difference of subscripts on bar determines that the values differ significantly at *P* > 0.05 level in all three generations like T_0_, T_1_, and T_2_ (2009–2011) and ginning out turn percentage statistical analysis represented Least Significant Difference (LSD) value 0.003 was greater than all difference of mean values from control. The difference of subscripts on bar determines that the values differ significantly at *P* > 0.05 level in all three generations like T_0_, T_1_, and T_2_ (2009–2011).

### Progress in agronomic characteristics of cotton

**Figure 9:**
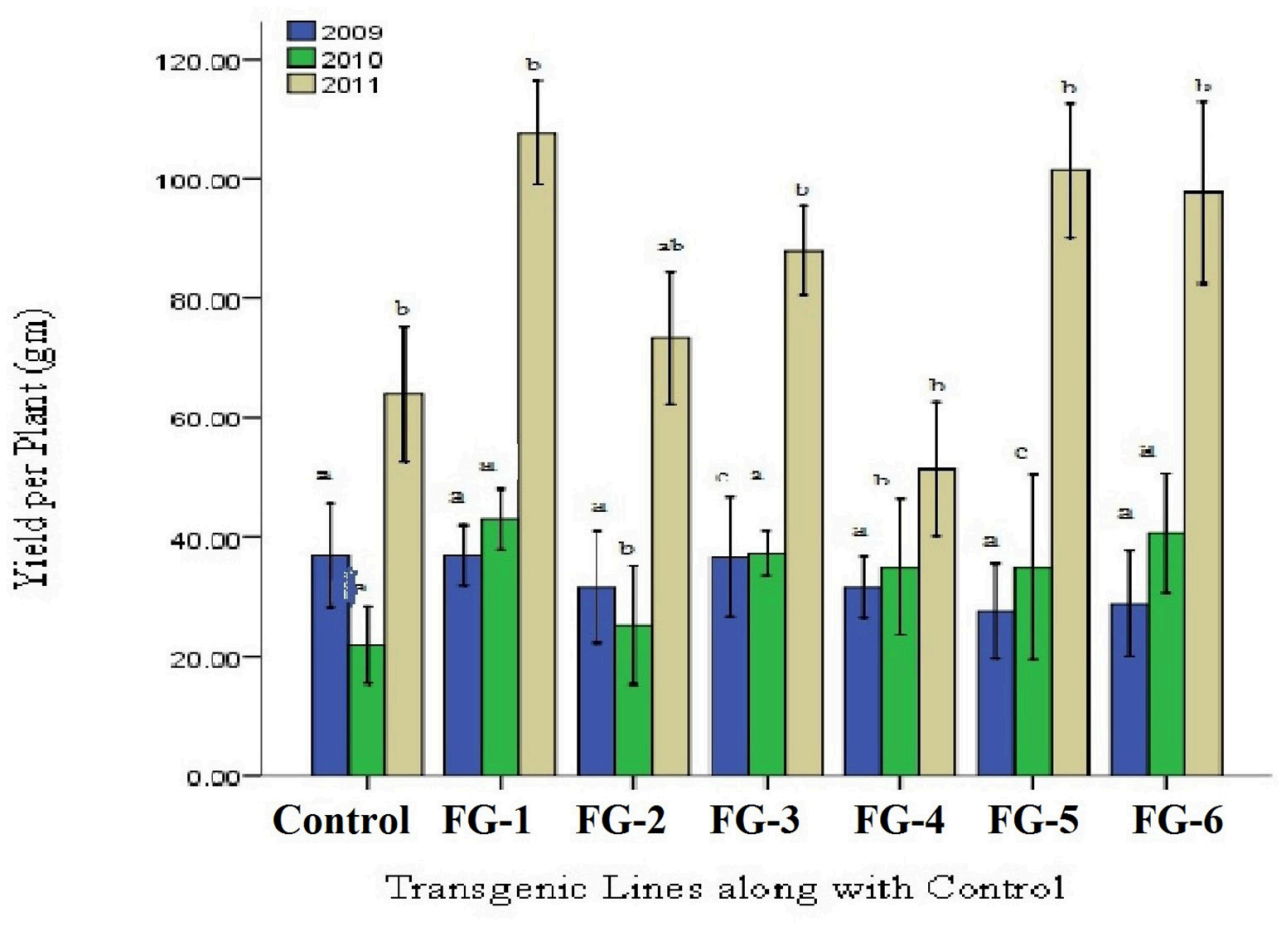
**Comparison of yield per plant of transgenic and control lines (T_0_, T_1_, and T_2_ Generations)**. Lane 1: Control Plant, Lane 2–7: yield per Plant of Different *GhEXPA8* transgenic cotton Plants, blue color represent yield analysis at T_0_ generation, green color indicated yield analysis at T_1_ generation, brown color highlight yield analysis at T_2_ generation, Note: Bar determined the standard error.

Increase in yield was the final ambition of plant transformation. Data revealed that with increasing fiber qualitative characteristics was correlated with improvement of plant yield. In FG-8-1, FG-8-13, and FG-8-15 average increase in yield was found to be 37.09% as compared to control (Figure 9). On an average 35% increase in yield of transgenic cotton plants with *GhEXPA8* fiber gene as compared to control which shows significant improvement in yield. Least Significant Difference values 0.376 (2009) was less than all difference of mean values from control but LSD value 0.008 (2010), 0.011 (2011) were greater than all difference of mean values from control. The difference of subscripts on bar determines that the values do not differ significantly in T_0_ generation (2009) and differ significantly at *P* > 0.05 level in T_1_ and T_2_ generations (2010, 2011). The LSD was performed to evaluate the morphological characters of transgenic cotton plants.

### Phenotypic and genotypic correlation coefficient

Statistical analysis was done for the confirmation of improvement of qualitative and quantitative characteristics of transgenic cotton plants. Field data of six transgenic cotton plants with *GhEXPA8* fiber gene was used for the analysis of phenotypic and genotypic correlation analysis. On the base of statistical analysis, performance of GhEXP A8-1, GhEXP A8-6, and GhEXP 8A-4 was better as compared with other genotypes for fiber strength, fiber micronaire value and fiber uniformity index (Figure S1, Table 2). It was persuaded from Table 3 that significant genotypic correlation was found for plant height with sympodial branches, yield per plant, boll weight and GOT at genotypic level but negative and significantly correlated with sample weight and lint weight.

**Table 2:** **Genotypic (G) and Phenotypic (P) correlation matrix for quality traits (fiber length, fiber strength, and fiber micronaire value and fiber uniformity index) of transgenic cotton**.

**Traits**	**r**	**Fiber strength**	**Fiber length**	**Fiber uniformity index**
Fiber Micronaire value	G	−0.46941^*^	−0.38705	−0.39151
Fiber Strength	G		0.683212^*^	0.744647^*^
Fiber Length	G			0.996888^*^
Fiber Micronaire value	P	−0.45977^*^	−0.37629	−0.38512
Fiber Strength	P		0.651721^*^	0.710354^*^
Fiber Length	P			0.968771^*^

*r, correlation; G, genotypic; P, phenotypic*.

* *Correlation for improvement of fiber characteristics on the base of gene expression in transgenes (cotton plants) and on the base of phenotypic (field performance) of transgenes related to fiber characteristics. Correlation between the impact of one fiber improvement character on other characters*.

**Table 3:** **Genotypic (G) and Phenotypic (P) correlation matrix for yield traits of transgenic cotton**.

**Traits**	**r**	**Average Monopodial branches**	**Average Sympodial branches**	**Average no. of bolls per plant**	**Average boll weight**	**Average yield/plant**	**Sample weight**	**Lint weight**	**G.O.T. %**
Average height	G	−0.24981	0.617031^*^	0.292504	0.411842^*^	0.559334^*^	−0.94807^*^	−0.9215^*^	0.832426^*^
Average Monopodial branches	G		0.891566^*^	0.289238	0.161237	0.461808^*^	0.331046^*^	0.339501	−0.16455
Average Sympodial branches	G			0.448371^*^	0.239605	0.684499^*^	−0.01446	0.000485	0.057627
Average no. of bolls per plant	G				−0.25991	0.511455^*^	−0.38614	−0.45438^*^	−0.17541
Average boll weight	G					0.623542^*^	−0.21579	−0.1214	0.733398^*^
Average yield/plant	G						−0.46003^*^	−0.41887	0.553421^*^
Sample weight	G							0.993722^*^	−0.70226^*^
Lint weight	G								−0.62032^*^
Average height	P	0.43902^*^	0.70431^*^	0.233217	0.21122	0.350392^*^	−0.00123	−0.02243	0.56634^*^
Average Monopodial branches	P		0.49123^*^	0.234128	0.10127	0.461624^*^	0.64217^*^	0.01941	−0.00345
Average Sympodial branches	P			0.523071^*^	0.20305	0.947314^*^	−0.00126	0.04015	0.003232
Average no. of bolls per plant	P				−0.20181	0.413451^*^	−0.01344	−0.01471	−0.11239
Average boll weight	P					0.461872^*^	−0.29123	−0.01483	0.52628^*^
Average yield/plant	P						−0.01281	−0.56322^*^	0.542621^*^
Sample weight	P							0.783241^*^	−0.00122
Lint weight	P								−0.04213

*r, correlation; G, genotypic; P, phenotypic; G.O.T, ginning out turn*.

* *Correlation between different morphological characteristics e.g., what ration of improvement in one character showed impact on other agronomic characteristics*.

## Discussion

Cotton is important for worldwide economies and, as the foremost natural fiber, is necessary for basic textiles. Cotton fiber is one of the most integrated and prevalent economic feature. Two methods to improve cotton fiber quality include traditional breeding methods and molecular genomic modification. Molecular approaches may be more successful to genetically transform desired characteristics, such as improved fiber quality and yield, rather than conventional breeding methods (Arpat et al., 2004; Wilkins and Arpat, 2005).

Recent genomic studies have advanced our understanding of cotton fiber development stages and signaling pathways (Walford et al., 2011). The mechanisms of cotton fiber development are complex because of the many signal transduction and transcriptional regulation components involved in the process (Lee et al., 2007). The cotton plant is a model organism for understanding the mechanisms of cell delineation and elongation (Ruan et al., 2000, 2001), cellulose synthesis (Haigler et al., 2001), and the connections between fiber and embryonic tissues in seeds (Han et al., 2013). Numerous studies on cotton fiber cell development have implicated plant candidate gene families were act as a critical regulators of boll and fiber development (Tan et al., 2013). During the cotton fiber development there are different types of candidate gene families that control the biochemical processes of fiber like xyloglucan endotransglucosylase hydrolase (GhXTH), brassinosteroids receptors (GhBRI1), and brassinosteroid dependent transcription factors (GhEER1, BZR1), RNG finger proteins (GhSNA1, BRH1), actin binding protein (GhGLP1), and gibberellic acid receptor (GhGD1, SLR1; Lee et al., 2007).

The genetic transformations of cotton fiber genes are directly correlated with improved crop yield. *Agrobacterium*-mediated transformation of cotton has been limited to the specific cultivars that can be regenerated in tissue culture (Zhang, 2012). Primarily interested in the transgenic approach, we generated cotton plants that overexpressed the fiber gene *GhEXPA8*. Ten local cotton varieties were tested for germination experiment, but only *G. hirsutum* var. NIAB 846 was suitable for transformation because of its higher germination percentage and low fiber quality. We determined whether the transformation of this gene into cotton improved its fiber properties as done by Bajwa et al. (2013).

To integrate the fiber gene *GhEXPA8* into *G. hirsutum* var. NIAB 846, we used *Agrobacterium*-mediated transformation (Figure 1). A total of 8500 embryos were isolated for the transformation of *GhEXPA8*, and we obtained 106 putative transgenic cotton plants for an efficiency of 1.24%. PCR analysis determined 6 out of the 106 putative transgenic cotton plants were positive for the *GhEXPA8* fiber gene (Figure 2) and were used for our experiments. These transgenic cotton plants were also confirmed by using marker genes primers such as 35S, VirG gene and NPTII selection marker gene as represented in Figure 3. These results were correlated with other scientist reported work such as Mawgood et al. (2010), Suratman et al. (2013), and Bajwa et al. (2014).

Southern blot analysis and copy number was used to confirm the integration of the fiber gene *GhEXPA8* in six transgenic plants and absence of the transgene in the other plants that were tested (Figure 4). Plant lines 1(*GhEXPA8*-1), 2 (*GhEXPA8*-4), 5 (*GhEXPA8*-13), and 6 (*GhEXPA8*-15) integrated two copies of *GhEXPA8* into their genome, and plant lines 3 (*GhEXPA8*-5) and 4 (*GhEXPA8*-11) integrated three copies of the target gene into their genome. The expression levels of 1 (*GhEXPA8*-1), 2 (*GhEXPA8*-4), 5 (*GhEXPA8*-13), and 6 (*GhEXPA8*-15) in lanes 1, 2, 5, and 6 are much higher than the other *GhEXPA8* transgenic plants and control plants, possibly due to the copy numbers of the transgene, gene positional effects, gene insertion effects, internal cell programming, or other environmental factors (Southern, 1975). Similar results were reported by Cantsilieris et al. (2012). More than two copy numbers may cause gene silencing.

Real-time PCR analysis of transgenic plant lines 1 (*GhEXPA8-1*), 2 (*GhEXPA8-4*), 5 (*GhEXPA8-13*), and 6 (*GhEXPA8-15*) indicated that these lines have higher mRNA expression of *GhEXPA8* than the control and other transgenic plant lines, in which mRNA expression was quite low (Figure 5). These results were correlated with the results of Yi and Hong (2012). There was greater than 60% improvement in cellulose content of transgenic plant lines 5 (*GhEXPA8-13*) and 6 (*GhEXPA8-15*) compared to control plants. Transgenic plant lines 1 (*GhEXPA8-1*) and 2 (*GhEXPA8-4*), respectively produced 50% and 20% more cellulose than the control plants, as shown in Figure 6. Similar results of improvement of cellulose content of transgenic cotton plant were presented by Li et al. (2015), Wang et al. (2009), and Teixeira et al. (2010).

The qualitative analysis that was performed on the transgenic plants included fiber length, micronaire values, and strength and uniformity index. The fiber uniformity indexes of the transgenic lines were not consistent across the three generations (2009–2011). The transgenic cotton lines for fiber uniformity indexes only increased 10% (Figure 7D). Figure 7B illustrate the improvement in fiber strength (17%). Similar results of improvement in fiber strength were also reported by Jiang et al. (2000). All of the transgenic plants showed improvement in fiber length (20%) and micronaire value (19.10%) with consistently higher fiber lengths and micronaire values in each generation (Figures 7A,C), which has been observed in previous studies (Luo et al., 2007; Machado et al., 2009; Zhang et al., 2010; Qin and Zhu, 2011; Li et al., 2015).

Based upon fiber analysis, we conclude that the transgenic plants expressing the fiber gene *GhEXPA8* had large improvements in fiber length and micronaire value (mike value) after genetic modification. Our results indicate an indirect correlation between fiber length and micronaire values. Other qualitative fiber characteristics of the transgenic plants improved in one generation but not in the following generation. These results correlate with previous studies (Li et al., 2005; Pu et al., 2008; Wang et al., 2009). Morphological characteristic analysis also indicated that this gene had a significant effect on ginning out turn percentage, an essential parameter for textiles (Figures 8A,B, 9).

It was persuaded from Figure S1 that the performance of GhEXP A8-1, GhEXP A8-6, and GhEXP A8-4 was better as compared with other genotypes for fiber strength, fiber micronaire value, and fiber uniformity index. It was suggested that the transgenic GhEXP A8-1 and GhEXP A8-6 may be used for the development of higher yielding and good quality cotton varieties. It was found from Table 3 that the performance of GhEXP A8-1, GhEXP A8-6, and GhEXP A8-5 was better as compared with other genotypes for monopodial branches, sympodial branches, plant height, boll weight, and number of bolls per plant. The Table 3 showed that the performance of GhEXP A8-1 and GhEXP A8-6 was better for yield per plant, GOT, lint weight and sample weight. The performance of GhEXP A8-1 and GhEXP A8-6 transgenic lines was very good for all studied traits as compared with other transgenic lines. The results showed that the selection for the improvement of yield and quality of cotton genotypes may be made from *GhEXPA8*-1 and *GhEXPA8*-6. The results from Table 2 indicated that positive and significant genotypic and phenotypic correlation was found between fiber strength fiber length and fiber uniformity index while negative and significant genotypic and phenotypic correlation was recorded between fiber micronaire value and fiber strength. Positive and significant genotypic and phenotypic correlation suggested that increase in the fiber length may have an important and significant effect on fiber uniformity and fiber strength. It was suggested that selection of transgenic lines on the basis of fiber strength and uniformity may be helpful to improve fiber quality and yield in cotton. The results were in accordance with the finding reported by Abbas et al. (2013, 2014).

On the base of genotypic and phenotypic analysis of agronomic characteristics, plant height was significantly correlated with monopodial branches, sympodial branches, yield per plant and GOT at phenotypic level. Monopodial branches were significantly correlated with sympodial branches, yield per plant and sample weight at genotypic and phenotypic levels. Sympodial branches were significantly correlated with plant height, monopodial branches, number of bolls per plant, and yield per plant at genotypic and phenotypic levels. Yield per plant was significantly correlated with plant height, monopodial branches and sympodial branches, number of bolls per plant, and GOT at genotypic and phenotypic levels. Lint weight was positively and significantly correlated with sample weight both at genotypic and phenotypic levels. Significant and positive genotypic correlation indicated that selection of higher yielding genotypes may be helpful to improve yield and quality of cotton genotypes (Tables 2, 3). The increase in the traits may be fixed on the basis of positive genotypic correlations. Negative and significant correlations suggested that the decrease in the trait may be fixed in next generations. The present results reflected the findings reported by Abbas et al. (2014).

The results of present study were suggest that over expression *GhEXPA8* fiber expansin gene was primarily improve fiber lengths and micronaire values (mike values), compared to other fiber qualitative characteristics. The results also suggest that expression of the *GhEXPA8* gene in cotton could be a useful method to genetically engineer cotton for higher fiber quality.

## Conclusion

The results of the present study support the hypothesis that increased expression of *GhEXPA8* in transgenic cotton plants led to improved fiber characteristics of cotton plants, as determined by molecular analysis. The results show that transgenic plants express more *GhEXPA8* than control cotton plants, which ultimately affects the quality of cotton fiber, including fiber length. Increased cotton fiber length may be valuable for the textile industry of Pakistan.

## Author contributions

KB has designed and conducted the experiments; the work was executed in the supervision of AS, Analysis of data and writing of manuscript was done by AR, Development of strategy like synthesis of construct was done by AB and AA. Proof reading, editing and final approval of the version to be published was done by TH.

### Conflict of interest statement

The authors declare that the research was conducted in the absence of any commercial or financial relationships that could be construed as a potential conflict of interest.

## Supplementary material

Supplementary Figure 1 **Statistical analysis of fiber strength, fiber micronaire value, and fiber uniformity index with the use of Principal Component biplot**.

Supplementary Table 1 **Primers for analysis of transgenic cotton plants**.
