# Supplementary figures and images for: Stable transformation and expression of GhEXPA8 fiber expansin gene to improve fiber length and micronaire value in cotton
Source: Front Plant Sci. 2015 Oct 31;6:838. doi: 10.3389/fpls.2015.00838 (PMC4628126; doi:10.3389/fpls.2015.00838)

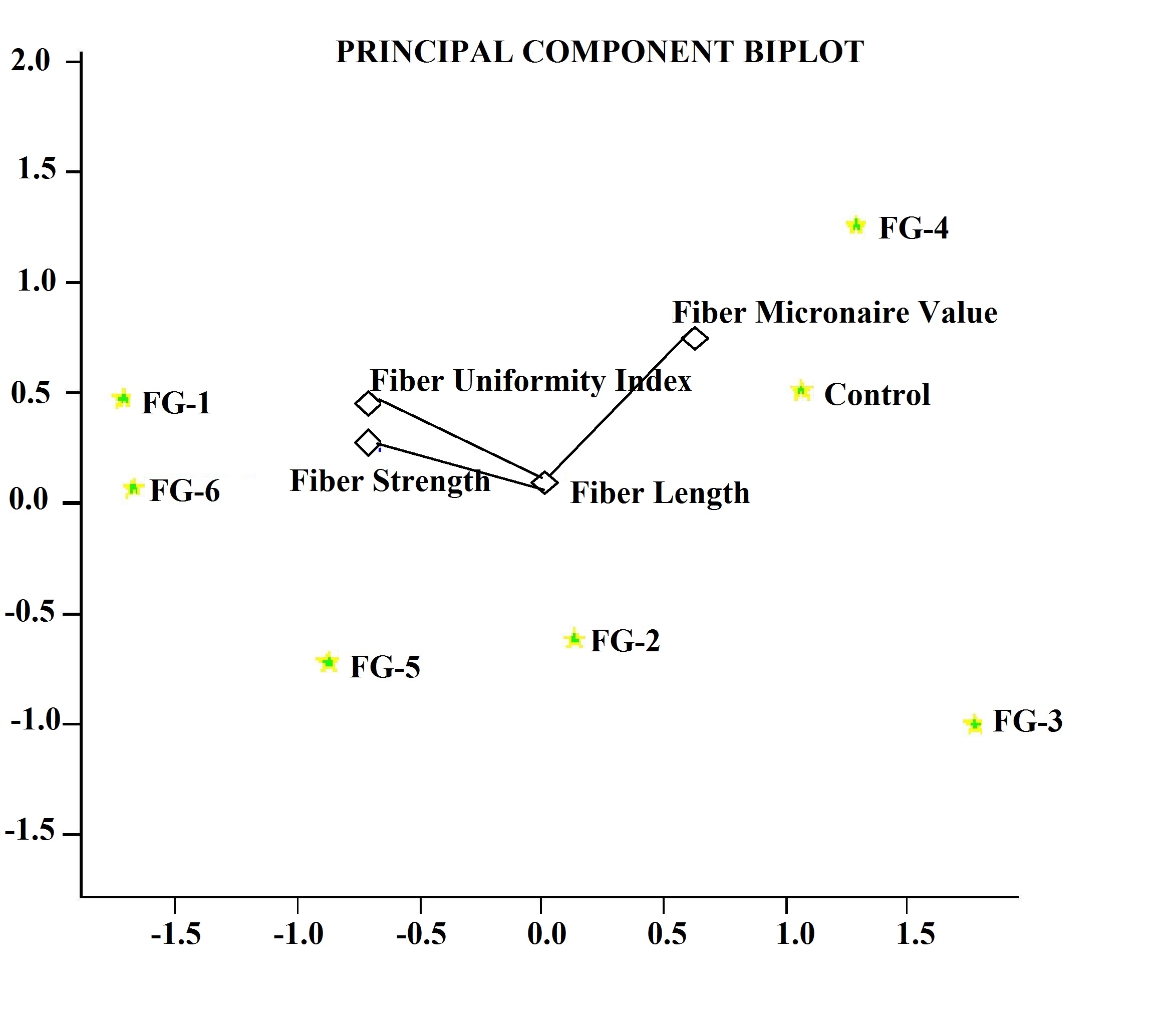

Supplement: Supplementary Figure 1 — Statistical analysis of fiber strength, fiber micronaire value, and fiber uniformity index with the use of Principal Component biplot. [file Image1.JPEG]
