# Supplementary material for: Stable transformation and expression of GhEXPA8 fiber expansin gene to improve fiber length and micronaire value in cotton
Source: Front Plant Sci. 2015 Oct 31;6:838. doi: 10.3389/fpls.2015.00838 (PMC4628126; doi:10.3389/fpls.2015.00838)
Supplement: Supplementary Table 1 — Primers for analysis of transgenic cotton plants. [file Table4.DOC]

| **Title** | **Forward primer** | **Reverse primer** |
| --- | --- | --- |
| **T_0_ generation (GhEXPA8)** | 5’-TGTGAGTAGTTTCCCGATAA-3’ | 3’-ATCCTTCCTTGTCTTCCTC-5 |
| **T_1_ and T_2_ generations**  **(GhEXPA8)** | 5’-CCCCCTAACTATGCTTTATC-3’ | 3’-ATTTGTAGAGAGAGACTGGTGA-5’ |
| **NPTII**  **(GhEXPA8)** | 5´-GAATGAACTGCAGGACGAG-3 | 3´-AGGTGAGATGACAGGAGATC-5’ |
| **35S CaMV**  **(GhEXPA8)** | 5´-AAGCCGTTCAGTATCAGAGA-3 | 3´-ATCCTTCCTTGTCTTCCTC-5’ |
| **VirG**  **(GhEXPA8)** | 5´-AAGCCGTTCAGTATCAGAGA-3’ | 3´-ACTCCTCAGTCACCCTTTG-5’ |
| **qRT-PCR**  **(GhEXPA8)** | 5’-GGTGCATTAGTCGAACCAT-3’ | 3’-GACGATCCCAGCTCGATATT-5’ |

**Primers for analysis of transgenic cotton plants**
